# Delayed inflammatory reaction to hyaluronic acid filler following Shingrix and Fluzone vaccines treated with lisinopril

**DOI:** 10.1016/j.jdcr.2022.03.018

**Published:** 2022-04-01

**Authors:** Jacqueline Witt, Deirdre Hooper, Girish Gilly Munavalli

**Affiliations:** a Department of Dermatology, Tulane University, New Orleans, Louisiana; b Dermatology, Laser and Vein Specialists of the Carolinas, Charlotte, North Carolina

**Keywords:** angiotensin II, angiotensin-converting enzyme inhibitor, delayed inflammatory reaction (DIR), dermal filler, hyaluronic acid, influenza vaccine, subclinical granuloma, varicella-zoster vaccine, ACE, angiotensin-converting enzyme, DIR, delayed inflammatory reaction, HA, hyaluronic acid, Th1, T helper type 1 cell

## Introduction

We present a case of a delayed inflammatory reaction (DIR) to Juvéderm Volbella and Juvéderm Voluma (Allergan) following the administration of Shingrix (GlaxoSmithKline) and Fluzone (Sanofi Pasteur) vaccines. The patient was successfully treated with low-dose lisinopril.

## Case report

**Fig 1:**
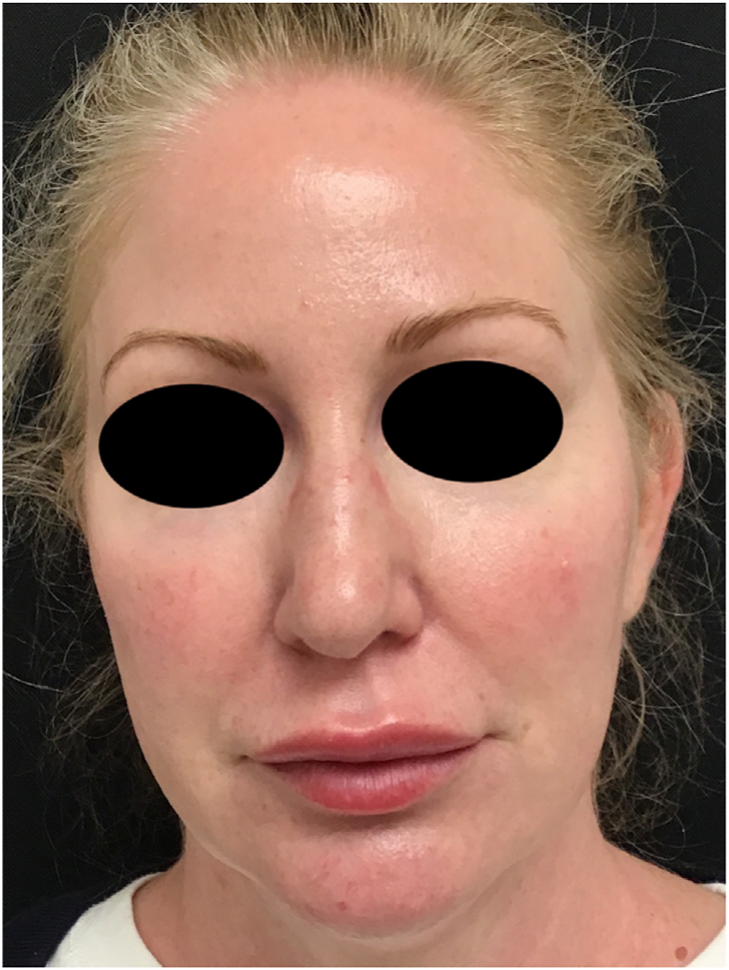
Image taken 24 hours after the patient developed swelling of her upper and lower cutaneous and mucosal lips.

**Fig 2:**
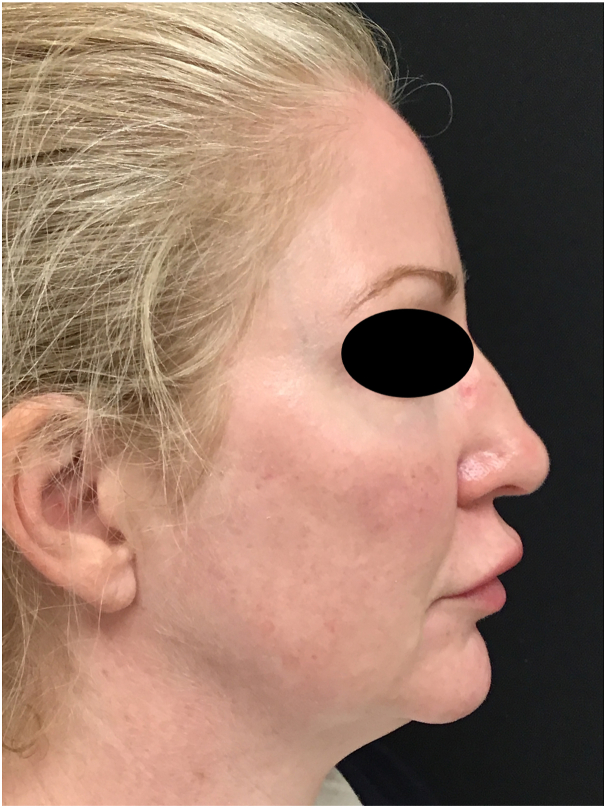
Image taken 24 hours after the patient developed swelling of her upper and lower cutaneous and mucosal lips.

A 52-year-old woman with psoriasis/psoriatic arthritis presented with a 2-day history of swelling of the lips and cheeks (Figs 1 and 2). Thirty hours prior to onset, she had received the Shingrix and Fluzone vaccines. Previously, she had been treated with Volbella to the lips (4 months) and Voluma to the cheeks (8 months). Before presenting, she had taken 25 mg diphenhydramine, but the swelling had worsened. She denied any history of drug allergies or angioedema. Her psoriasis/psoriatic arthritis was well controlled with adalimumab, which had last been administered 10 days before vaccination. Her medical history was significant for hypothyroidism (treated with 50 mcg levothyroxine per day), depression (450 mg bupropion per day), and anxiety (15 mg buspirone twice daily). She denied recent dental work. Over the past 6 years, she had been treated with Volbella, Juvéderm Ultra (Allergan), and Voluma multiple times, without any adverse events. Most recently, Volbella had been injected to the lips (4 months), Voluma to the cheeks (8 months), and Ultra to the lips and nasolabial folds (20 months).

**Fig 3:**
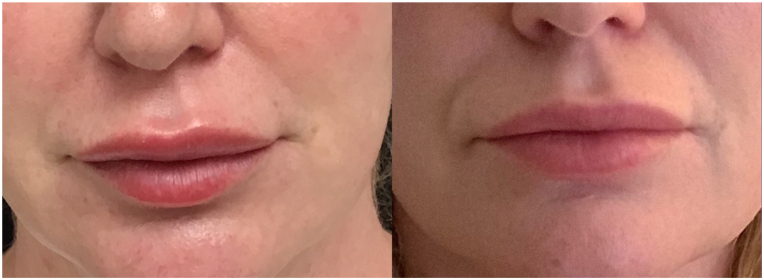
Comparison of lip swelling prior to treatment (*left image*) with resolution of swelling 16 hours after 1 dose of 5 mg lisinopril (*right image*).

She declined treatment with prednisone (due to the concern for side effects) and hyaluronidase (because she did not want to dissolve her filler). She was prescribed 5 mg lisinopril and reported the complete resolution of swelling 16 hours after 1 dose (Fig 3). She continued to take 5 mg of lisinopril daily for an additional 2 days, with no additional or rebound symptoms.

## Discussion

DIRs to hyaluronic acid (HA) fillers are rare and have been reported in association with influenza-like illnesses, infections, and dental surgeries and following COVID-19 vaccines.^1^, ^2^, ^3^ These reactions are thought to be type IV delayed hypersensitivity reactions and occur days to months after filler placement. A DIR may present with swelling at the site of the injection or the development of painful granulomas, with an estimated incidence of 0.5% within 5 years of treatment with HA filler.^4^ There has been an increase in reports of DIR in patients with HA fillers since COVID-19 vaccines were released in 2020, which has led to growing interest in this poorly understood phenomenon.^3^

A recent, novel case series reported the efficacy of lisinopril for the treatment of COVID-19 vaccine–related DIR to HA fillers.^3^ The COVID-19 messenger RNA within the SARS-CoV-2 messenger RNA vaccine induces the production of SARS-CoV-2 surface spike proteins within the host.^5^ The spike proteins irreversibly bind to the membrane-bound angiotensin-converting enzyme (ACE)2 receptors, leading to the inability to control angiotensin II and upregulation of the proinflammatory cascade.^6^ The authors proposed that blocking the conversion of angiotensin I to angiotensin II with an ACE inhibitor blunts the proinflammatory cascade, leading to the resolution of the hypersensitivity reaction to the HA filler. The authors also hypothesized that increased levels of angiotensin II from SARS-CoV-2 vaccination may stimulate quiescent subclinical granulomas formed by the breakdown of HA fillers, generating a T helper type 1 cell (Th1)–driven immune response and a DIR.^3^

Our case demonstrated the improvement of a DIR to Juvéderm Volbella and Voluma that occurred after Shingrix and Fluzone vaccines and was successfully treated with 5 mg lisinopril. Lisinopril is an ACE inhibitor that was approved by the Food and Drug Administration in 2003 for the treatment of hypertension. Lisinopril is typically well tolerated and has a half-life of 12.6 hours.^7^^,^^8^ Shingrix is an inactivated recombinant vaccine with the varicella-zoster virus glycoprotein E as its antigen. Fluzone is an inactivated quadrivalent influenza vaccine with influenza hemagglutinin surface protein as its antigen.

We propose that ACE inhibitors cause a shift in the natural proinflammatory cascade mediated by the renin-angiotensin-aldosterone system. By blocking the production of angiotensin II, there is a decrease in the Th1-mediated reactivation of subclinical granulomas formed by the degradation of the HA filler. Angiotensin II has been shown to activate a downstream pathway leading to the differentiation of naïve helper T cells into Th1 cells via signal transducer and activator of transcription 1 and nuclear factor kappa light chain enhancer of activated B cells, which are known to play a role in granuloma formation.^9^ Immune stimulation from inoculation or other stressors leads to the body’s natural response to shift to a proinflammatory cascade. The mechanism behind the increase in DIR following SARS-CoV-2 vaccinations is likely twofold; the spike protein increases the production of angiotensin II, and the natural Th1 immune response of immunization leads to a reactivation of subclinical granulomas.

Our patient did not develop any reactions following 2 doses of the Pfizer BNT162b2 SARS-CoV-2 vaccine 1 year after treatment of her cheeks with Voluma and lips and nasolabial folds with Ultra, nor did she have any reaction to a booster Pfizer vaccine 2 months after her lips were treated with Volbella. We suspect that the patient’s use of a tumor necrosis factor inhibitor would not blunt the DIR, as the angiotensin II pathway would still result in the upregulation of CD44, the homing signal for the immune response to free short-chain HA.^3^ One may speculate that the patient’s psoriasis could elevate her risk of DIRs given the increase in Th1 cells seen in psoriasis.

Prior to the patient starting lisinopril, we reviewed her comorbidities and medications, with close attention to any history of hypotension, hypertension, or hyperkalemia. We start 5 mg lisinopril daily in patients with hypotension. If a patient is normotensive or hypertensive, we start 10 mg lisinopril daily. This dose is continued for 3 days, typically with the resolution of swelling early in the treatment. If there is no improvement after 1 day of 5 mg lisinopril, the dose is increased to 10 mg daily. We have used this dosing regimen prior to immunizations in patients who had previously experienced DIRs after immunizations.

To our knowledge, this is the first reported case of Shingrix or Fluzone vaccine–induced DIR to HA fillers to be treated with lisinopril. We recognize that it is difficult to determine whether the response was due to lisinopril, but given that the patient was progressing at presentation, we suspect that the lisinopril played a role in her rapid improvement. We propose that the treatment of DIRs with lisinopril should be considered and that additional studies examining the role of the renin-angiotensin-aldosterone system in inflammatory conditions is warranted.

## Conflicts of interest

Dr Hooper is an investigator and consultant for Allergan and Galderma. Dr Munavalli is an investigator and consultant for Allergan. Dr Witt has no conflicts of interest to declare.
